# Personalization of medical treatments in oncology: time for rethinking the disease concept to improve individual outcomes

**DOI:** 10.1007/s13167-021-00254-1

**Published:** 2021-10-07

**Authors:** Mariano Bizzarri, Valeria Fedeli, Noemi Monti, Alessandra Cucina, Maroua Jalouli, Saleh H. Alwasel, Abdel Halim Harrath

**Affiliations:** 1 Department of Experimental Medicine, Systems Biology Group Lab, University La Sapienza, via Scarpa 16, 00160 Rome, Italy; 2 Azienda Policlinico Umberto I, Viale del Policlinico 155, 00161 Rome, Italy; 3 Department of Surgery “Pietro Valdoni”, Sapienza University of Rome, 00161 Rome, Italy; 4 Department of Zoology, College of Science, King Saud University, Riyadh, 11451 Saudi Arabia

**Keywords:** Predictive preventive personalized medicine (PPPM), Systems biology, Polypharmacology, Critical transitions

## Abstract

The agenda of pharmacology discovery in the field of personalized oncology was dictated by the search of molecular targets assumed to deterministically drive tumor development. In this perspective, genes play a fundamental “causal” role while cells simply act as causal proxies, i.e., an intermediate between the molecular input and the organismal output. However, the ceaseless genomic change occurring across time within the same primary and metastatic tumor has broken the hope of a personalized treatment based only upon genomic fingerprint. Indeed, current models are unable in capturing the unfathomable complexity behind the outbreak of a disease, as they discard the contribution of non-genetic factors, environment constraints, and the interplay among different tiers of organization. Herein, we posit that a comprehensive personalized model should view at the disease as a “historical” process, in which different spatially and timely distributed factors interact with each other across multiple levels of organization, which collectively interact with a dynamic gene-expression pattern. Given that a disease is a dynamic, non-linear process — and not a static-stable condition — treatments should be tailored according to the “timing-frame” of each condition. This approach can help in detecting those critical transitions through which the system can access different attractors leading ultimately to diverse outcomes — from a pre-disease state to an overt illness or, alternatively, to recovery. Identification of such tipping points can substantiate the predictive and the preventive ambition of the Predictive, Preventive and Personalized Medicine (PPPM/3PM). However, an unusual effort is required to conjugate multi-omics approaches, data collection, and network analysis reconstruction (eventually involving innovative Artificial Intelligent tools) to recognize the critical phases and the relevant targets, which could help in patient stratification and therapy personalization.

## Introduction

Personalized medicine (PM) [1] — “to match the right drugs to the right patients” — has become a widely used term in both the scientific community as well as in the public debate, its vagueness notwithstanding. Indeed, PM lacks an unambiguous definition and is open to interpretation [2]. In the early twentieth century, PM was referring to a number of integrated medical resources set in place to address patient’s needs in a “holistic” way. As such, a “personalized approach” is a common tenet of old western practitioners and physicians in the eastern world, the latter mostly relying for their treatments on “personalized,” complex mixture of herbal compounds, assembled according to the specific traits of each individual [3]. However, in the last 20 years, PM acquired a different meaning, indicating a target-based treatment for selected sub-groups of patients carrying the genetic/biochemical abnormalities considered the driver cause of the disease under scrutiny. Unfortunately, this definition involves a number of unresolved epistemological and theoretical issues that further complicate an already complex puzzle. Just to start with, several illnesses — like cancer, cardiovascular diseases, and diabetes — cannot be ascribed to a unique, simple deregulated genomic pathway, while effective drug-based targeting of such processes is still far from being achieved with our current technologies. PM seeks to improve stratification and timing of health care by utilizing information primarily obtained from the lowest biological level, i.e., the genomic-proteomic level. Therefore, a major drawback of PM lies precisely on the fact that this approach disregards almost completely those factors acting at levels higher than the cellular one (microenvironment, tissue, and physiological levels), whose contribution is anything but irrelevant in triggering the transition from healthy status to disease.

Therefore, it is not so surprising that clinical studies were finally unable to substantiate the very preliminary expectancies of PM. In fact, most cancer patients clustered according to sophisticated genomic testing do not benefit from a “precision medicine” strategy [4, 5]. This finding, altogether with the epistemological indeterminacy that wraps the concept of PM, can partly explain why in the last years PM has lost much of its charm, as evidenced by a decrease in published papers, the US-2016 Precision Medicine Initiative and the Cancer Moonshot effort notwithstanding. Therefore, some authors have tried to overcome such hurdles by incorporating “extra-genomic” factors in the PM framework, thus implicitly recognizing the intrinsic inadequacy of the preliminary approaches [6], despite the field of PM has been extended to include objectives pertaining Predictive, Preventive and Personalized Medicine (PPPM/3PM).

## Promises and premises of personalized medicine

Advances in genomics have allowed to stratify patients into distinct groups, based on a few molecular differences, which are deemed to play a critical role in the pathogenesis process. According to these premises, disease individuation would principally rely on the gene-expression pattern associated in a specific patient, allowing depicting a one-to-one correspondence upon a hypothetical Cartesian space, between the “genomic signature” and the illness in each individual [7]. This Promethean dream comes true in 1999, when Francis Collins established in a seminal paper the ways the human genome would be used to predict, prevent, and treat disease in the next 10 years, so as a complete transformation in medical practice would be expected even before 2020 [8]. This is the (bewildering) promise. However, we are in 2020 and this astonishing revolution in medicine has not yet been glimpsed.

Clinical randomized trials have provided little if any evidence of benefits when patients with different diseases have been treated with personalized-based treatments [9, 10]. This failure has contributed to the emergence of the so-called “reproducibility crisis” in biology and medicine [11, 12]. Still worse, these disappointing results prompted to cast on doubt the foundational assumptions of precision medicine [13].

Indeed, even “simple,” monogenic diseases — in which a single point mutation is recognized as the “main” causative factor — are not “simple” in their pathogenesis, as a number of additional factors, distributed across different hierarchical levels of the living organisms (from the DNA to physiological apparatus), are ultimately responsible of the disease phenotype. A paradigmatic case is provided by sickle cell anemia, a classic monogenic disorder, in which the interplay among a number of context-dependent cues enacts the emergence of no less than six different pathological phenotypes [14]. Some patients develop principally painful crises with or without bony infarcts; others are prone to hemolytic emergencies; some develop vaso-occlusive crises, including stroke; still others develop acute chest syndrome, while many are phenotypically normal, except for mild anemia. What matters here is that the treatment — to be “precise” and “personalized” — must be tailored according to the emerging clinical pathophenotype, and not based on the primary point mutation. In this case, PM could hardly fit the specific needs of each patient, given that the genotype does not match with the (disease’s) phenotype [15]. Additionally, gene variants (including mutated genes), initially thought to play a “pathogenetic” role in several complex diseases, have been “reclassified,” given that their involvement becomes “problematic” [16]. Overall, in many pathological conditions, gene variance does not seem to play a relevant role, as the genes involved do not seem either to possess any biological link with the pathogenetic mechanism, or they can offer any clinical utility for prognosis [17]. As a fact, the relative risks for the vast majority of gene variants rarely exceed 1.5, and these variants have added little useful predictive power to traditional risk prediction algorithms. Even for diagnostic purposes, wide genome analysis often fails to equate the predictive power of classical medical parameters (anamnesis, neighborhood, socioeconomic status, dietary habits), and sophisticated assessment of gene-expression patterns adds little (if any) to conventional predictive models [18]. Consequently, the main obstacle to progress in PPPM approaches for cancer is the lack of validated prognostic and predictive biomarkers [19].

It has been argued that the discouraging results obtained by PM-based clinical trials should be attributed to selection bias and uncertainty in defining clear outcomes [20], while developing more sophisticated approaches to identify specific patients subsets — using broad molecular testing and integrated genomic data from liquid biopsy samples [21, 22] — would in principle help in overcoming such a failure. This is wrong, as although the number of patients eligible for genome-driven treatment has increased over time, these “tailored” drugs have helped only a minority of patients with advanced cancer [23]. Moreover, unambiguously statistical criteria for patient’s selection and outcome parametrization are still inadequate. Several technical methodologies — including unsupervised discovery and data mining — have been used without explicating clear hypotheses to justify the observed (statistical) correlations. Therefore, selection bias and factors that can distort exposure-outcome correlation are usually overlooked. However, population-based studies of a disease require specific theoretical assumptions that inform data collection and allow to ascertain both exposures and outcomes in a standardized fashion [24].

To address such issues, instead of reconsidering the biological assumptions on which the PM strategy has been developed, an increasing number of scientists preferred to bypass that hurdle by adopting a new statistical-biometric approach as such provided by the Big Data Theory. Namely, the development of omics and systems biology has promoted one to gradually change paradigms in oncology from traditional single-factor strategy to multi-parameter systematic strategy [25].

Yet even this framework showed to be unsuccessful [26]. Data handling does not produce any new information by itself, as correlation does not mean “causation.” Furthermore, few prognostic factors or systems are robustly validated, and still fewer have made a convincing difference in health outcomes or in prolonging life expectancy [27]. In most diseases and outcomes, a considerable component of the prognostic variance remains unknown for our understanding of the critical mechanisms on whom the disease process depends is still insufficient. Additionally, most correlations are spurious, i.e., very large databases likely contain arbitrary correlations [28]. Empowerment of statistical analysis and sophisticated modeling cannot compensate for the lack of theory into which information from experiments need to fit. Computationally intensive tools for the exploitation of huge data sets are still based on poorly designed model; presumptively, they can only help in generating new hypotheses, but not true explanations. Consequently, applications of Big Data Theory have met with limited success in scientific domains, up to now [29]. Thereby, a new theoretical framework is urgently warranted “as a guide to experimental design for maximal efficiency of data collection and to produce reliable predictive models and conceptual knowledge” [30].

## Inadequacies of theoretical assumptions at the root of the personalized medicine

Especially in the oncologist community, several scholars have enthusiastically welcomed personalized medicine (i.e., precision medicine) as a solution to find selective drugs able in modulating sensitive, key targets deemed to “drive” the overall process of tumor regression [31, 32]. Target-based therapies are broadly rooted into a theoretical framework as such provided by the Somatic Mutation Theory (SMT).

According to SMT, cancer is a cell-based disease [33], due to the accumulation of somatic mutations and/or chromosomal aberrations that alter the control of proliferation in a single cell that eventually will generate a neoplasia. This approach is essentially “reductionist” in essence, as it posits that the system — cells, tissues, cancer — can be explained by studying its parts in isolation, while the principal causative factor must be identified at the lowest level of organization (i.e., DNA, proteins, and so forth). This model has been extensively criticized given that is unable to accommodate with an increasing number of controversial and paradoxical results [34, 35]. For instance, accumulating evidence shows that genomic alterations, such as those in BRAF, RAS, EGFR, HER2, FGFR3, PIK3CA, TP53, CDKN2A, and NF1/2 genes — all of which are considered hallmark drivers of specific cancers — can also be identified in benign and premalignant conditions, occasionally at frequencies higher than in their malignant counterparts [36, 37].

Moreover, we usually forget that the search for critical genomic targets is seriously flawed from the outset by the unavoidable, intrinsic genomic heterogeneity of cancerous tissues [38]. Distinct mutations can be present as high as 100 million even in a single tumor [39], while sequencing and genome analysis of multiple biopsies from different regions of the same tumor reveals the wide spreading of genomic heterogeneity [40]. Intratumor heterogeneity is present since the early steps of cancer development and it is uncanny that chemotherapy can select further subclones, sharing increased aggressiveness and dedifferentiation, leading cancer to progressively becoming insensitive to any medical control [41]. Such heterogeneity yields a mosaic of different cells and, ultimately, can hamper cancer treatment [42]. This inescapable complexity contradicts the superficial textbook concept of clonal expansion of a dominant cell clone carrying the oncogenic mutation that “takes over” the entire tumor. In the real world, each cell shows a distinct set of mutations, while the subset of cells carrying “driver” mutations is unable to canalize the overall population into a unique, homogenous gene-expression pattern. In fact, although some tumors harbor a dominant population emerging from clonal selection, genomic diversity is the rule than the exception [43]. This process not only entails the topological distribution of cancer cells within the tumor, but also emerges at multiple time points during tumor progression, as demonstrated by liquid biopsies obtained from serial samples [44]. These data indicate that distinct clusters from the same tumor may undergo independent progression pathways, which culminate into the simultaneous presence of different phenotypic populations, each one harboring different malignant traits. This bewildering variety in cancerous phenotypes cannot be merely ascribed to differences in genomes but call into question the existence of epigenetic mechanisms and subtle modulation of the gene regulatory networks [45]. This non-genetic heterogeneity of tumor cell states defies a “precise” genotype–phenotype causal relationship and allows them to adapt to both environmental perturbations (nutrients availability, cells crowding, and hypoxia) as well as treatments without enacting the appearance of additional mutations. Again, these findings confirm that there is no straightforward linear causal relationship between tumor genotype and phenotype [46]. Moreover, cancer cells within individual tumors often exist in distinct phenotypic states. Given certain conditions, any subpopulation of cells (i.e., with different phenotypes) will return to equilibrium phenotypic proportions over time, after experiencing a critical transition [47], which enacted the disclosure of multiple, branching differentiating trajectories [48]. Notice that cancer stem-like cells arise de novo from non-stem-like cells, thus “regenerating” the malignant potentiality of the tumor. This is why seemingly identical cells respond differently to treatments, given that phenotypic and genotypic differences provide differentiated response by activating even opposite outcomes in cell behavior and ultimately escaping the drug-induced inhibition on specific targets [49]. Overall, these findings highlight how complex and unstable is the gene-expression pattern of a tumor population, within each patient [50].

This body of evidence cannot easily accommodate with the prevailing carcinogenesis model. Instead, accumulated pitfalls contribute to laying bare the inadequacy of the SMT. To overcome these limitations, SMT became subject to a number of course-corrections, which strive to integrate new concepts by recurring to twisted arguments [51]. Latest versions of SMT include even the microenvironment — viewed as instrumental in promoting carcinogenesis — while trying to preserve the native mutation-based hypothesis [52]. All in all, these attempts look like the epicycle-based strategy used in ancient time to accommodate with the experimental facts that challenged the Ptolemaic system [53]. In alternative, in order to get rid of these conundrums, cancer has been proposed as an emergent phenomenon, due to a deregulated cross talk between cells and their microenvironment [54]. Carlos Sonnenschein and Ana Soto have conceptualized this new framework within the Tissue Organization Field Theory (TOFT) [55, 56]. TOFT is anchored at the tissue level of biological organization and conceives the development of cancer as a relational problem, focusing not on a single cell type but, as in organogenesis, on the interactions among different cell types and their microenvironments. The alteration in the interplay among those components involves different levels of organization and a number of *secondary* changes, eventually including the emergence of disrupted gene-expression patterns. In agreement with TOFT, mutated genes are the result, and not the cause, of the disrupted normal tissue architecture that eventually ends up in fostering cancer onset [57]. Therefore, changes in genomic profiles or in biochemical pathways can only be “associated” rather than considered as “causative.” Admitting this bitter conclusion would deprive precision medicine in oncology of its rationale, given that PM relies on targeting a validated and genetically stable driver of disease. To date, proof-of-concept trials have not supported this premise [12, 58]. Moreover, target-based treatments in oncology suffer from two major drawbacks: (1) Currently available inhibitors of specific pathways provide only minimal or complete blockade of biochemical pathways and are therefore inefficient or too toxic to be used [59]. (2) Second, critical networks in cancer — as well as in living cells — show a bewildering plasticity and adaptability, even under harsh environmental conditions, thus allowing the system to escape from programmed cell death [60]. Overall, those considerations help in explaining the shift in interest from the cancer cell to the stroma [61] and substantiate the relevance of microenvironment-based studies in search of new treatment options, as advocated by TOFT [62].

Personalized treatments are unable to cope with such an overwhelming complexity [63], and the small improvement in cancer survival recorded in the last years can only minimally be ascribed to target-based therapy [64, 65]. Namely, randomized, large studies with different combinations of target-based treatments in a number of cancer types failed to demonstrate any significant encouraging efficacy in any of the treatment arms or patient subsets [66, 67]. For instance, a multicenter randomized trial of treatment based on tumor sequencing compared with conventional cancer treatment showed no advantage of sequencing [68], as did the NCI-MATCH (National Cancer Institute–Molecular Analysis), in which almost 6,000 patients have been enrolled [69]. Similarly, a basket trial testing molecularly guided treatment approaches for multiple mutations in advanced non–small-cell lung cancer was demonstrated to be unsuccessful [70]. Expectancies from the recently introduced immunotherapy approaches have been disappointing as well. Immunotherapy had only limited effects on the drop in overall cancer mortality. The undisputed benefits for melanoma and metastatic lung cancer are impressive, but so far they affect relatively few people and are associated with life-threatening side effects [71]. Overall, the weight of this evidence prompted to suggest that “our best weapons against cancer are not magic bullets” [72]. Therefore, precision medicine — especially in oncology — has lost most its fashion, while raising embarrassing concerns. A sober view of the evidence derived from prospectively designed trials of personalized medicine inevitably leads us to consider “that our current oncology community will be guilty of hubris and of overpromising what we can deliver in a realistic time line” [73].

## Disease: how to reframe the concept

It is disheartening that the debate on PM has left aside a preliminary premise: what do we mean by “disease”? [74] No doubt that a number of inconsistencies of our modern treatment strategies — including those claimed by PM — should be attributed to the controversial concept of human disease. The debate on that subject mostly developed around two opposite positions represented by Constructivism and Naturalism. The former essentially denies the naturalist thesis that disease necessarily encompasses bodily malfunction and claims for a general “reformation” of the concept of disease, conceived as a “societal,” historical-based construct [75]. On the contrary, according to the naturalistic perspective — by far the only to which the daily medical practice actually relies — a disease involves malfunction of organs/apparatus, which either can or cannot be perceived as such, i.e., by complaining symptoms [76].

The latter model originates from Virchow’s conclusion that all diseases result from cellular abnormalities [77]. Since the discovery of the double helix in the 50 s, however, that framework was strongly superseded by an even more reductionist approach, as that provided by the New Genetics, which posits that every disease can be traced back to the malfunctioning of a discrete number of genes [78]. Briefly, this reductionist model relies on the following three premises: (1) the disease recognizes a dominant (molecular) cause; (2) medical signs and symptoms — which altogether constitute the disease phenotype (the “pathophenotype”) — are linearly correlated with the molecular cause; (3) removal/correction of the underlying, putative “cause” will restore healthy conditions. Sad to say, that model still awaits to be vindicated beyond any reasonable doubt, especially for degenerative diseases or mental illness [79]. Moreover, such framework becomes problematic when considering in the perspective of “preventive” medicine [80]. Are presumptive markers of a “future” disease condition reliable enough to ask for a “preventive cure”? Could a genomic profile allow drawing a reliable probabilistic ascertainment of a future disease? And then, could someone with a “genetic predisposition” (whatsoever this really means) be considered already sick?

Nevertheless, in the last 30 years, diseases have been increasingly “equated” to the malfunctioning of a few, critical pathways or of their related driver genes. Consequently, drug discovery has been dominated by reductionism, aiming to identify drugs that activate or inhibit specific molecular targets. Unfortunately, therapeutic approaches based on such a simplistic paradigm often showed either unforeseen toxicity or lack of efficacy when tested in clinical trials [81].

Consequently, the adoption of this reductionist-based approach progressively distorted and shaped medical practice — namely by radically modifying the diagnostic methodology and the doctor–patient relationship — leading toward a new “disease taxonomy” [82]. Consequently, we are witnessing a number of inadequacies in the current medical practice, which often reflect a lack of specificity (i.e., inability in defining a disease unequivocally), and a lack of sensitivity (i.e., incapacity in recognizing preclinical, true causative state of disease). Ultimately, this model is proven to be confounding, as it often posits wrong correlations between the disease-associated biological parameters (usually identified only when illness reaches a “stable-state”) and the alleged causative processes, thereby prejudicing efficient treatment strategies.

At a first glance, the starting premise of PM relies on the following, abridged statement: one genotype-one pathophenotype. This is wrong, even for simple monogenic diseases. The abovementioned example of sickle cell anemia highlighted a complex interplay of different causative factors — spanning from cell to higher levels of living system’s organization — which ultimately fosters the emergence of no less than six different disease phenotypes. Similarly, EBV infection is recognized to promote mononucleosis or Burkitt’s lymphoma.

In both situations, B cells represent the primary target of EBV [83]. In Western countries and in presence of well-regulated immune system, the virus-induced proliferation of B cells is intrinsically controlled by CD8 + and CD4 + T cells. In this setting, the EBV infection generally triggers infectious mononucleosis. On the contrary, in inhabitants of the several equatorial regions of the world, EBV induces the development of lymphoid tumors [84]. The difference lies on the previous medical history of the host and on the specific interactions that EBV triggers with the immune system of each individual [85]: a set of factors that cannot be deduced from the genomic analysis neither of the viral genome, nor the genome of the host. In simple words, the specific disease arises from a dynamic process in which the primary causative factor (the virus) interacts in a non-linear fashion with complex apparatus of the host (not limited to the cellular compartment); organs, cells, and tissues are in turn “shaped” and “customized” by the previous medical history of the organism living in a very unique environment [86]. As a result, EBV infection is currently known as the main “causative” factor of a number of disparate diseases, including pharyngeal carcinomas [87], gastric cancer [88], and non-malignant illness, such as the childhood disorders of Alice in Wonderland Syndrome [89], systemic lupus erythematosus [90], and acute cerebellar ataxia [91]. This example epitomizes that a disease cannot be considered a “static” state but should instead be considered a dynamic process, ruled by non-linear dynamical relationships, which can ultimately drive the system toward very different outcomes. Broadly speaking, “genetic defects” cannot predict the pathophenotype, which ultimately emerges from the complex interactions among different factors, distributed across several, hierarchically organized levels.

According to the PM premises, the causative factor(s) that are thought to contribute to the disease process still should be “at work” at the time of treatment. However, as happen for several conditions, the mechanism/gene responsible for the onset of the illness might have exerted its action during early pathogenic steps and could no longer be active during the steady state of the disease, when diagnosis is usually reached. Some developmental-based diseases, like mental illness [92]or cardiovascular diseases, fall within this category, as well as some cancers that “must” lose their mutated “driver” oncogenes precisely when they metastasize and — paradoxically — become more aggressive [93].

However, pathogenic interactions are distributed across a space-temporal continuum, given that a number of genomic-related factors are likely to act only during some critical developmental phases (during the intra-uterine life, at the birth, in the neonatal, and pre-pubertal period). Therefore, disease development is a time-dependent process, tightly linked to the patient’s history. In the last resort, disease should be viewed as a manifestation of developmental plasticity, the phenomenon by which one genotype can give rise to a range of different physiological or morphological states in response to different environmental conditions during development [94]. Therefore, numerous chronic diseases are currently supposed to arise from some “disturbances” acquired during critical developmental periods [95].

Epigenetic and post-translational changes can efficiently begin as early as during pregnancy (still in the womb) and may be affected by the paternal/and maternal environments, as well as by early life events (dietary habits, childhood diseases, premature exposure to environmental carcinogens and toxicants like endocrine disruptors) [96]. These modifications play a critical role in shaping cells and tissue sensitivity to carcinogens, ultimately favoring the emergence of cancer in the adult life [97, 98].

**Fig. 1:**
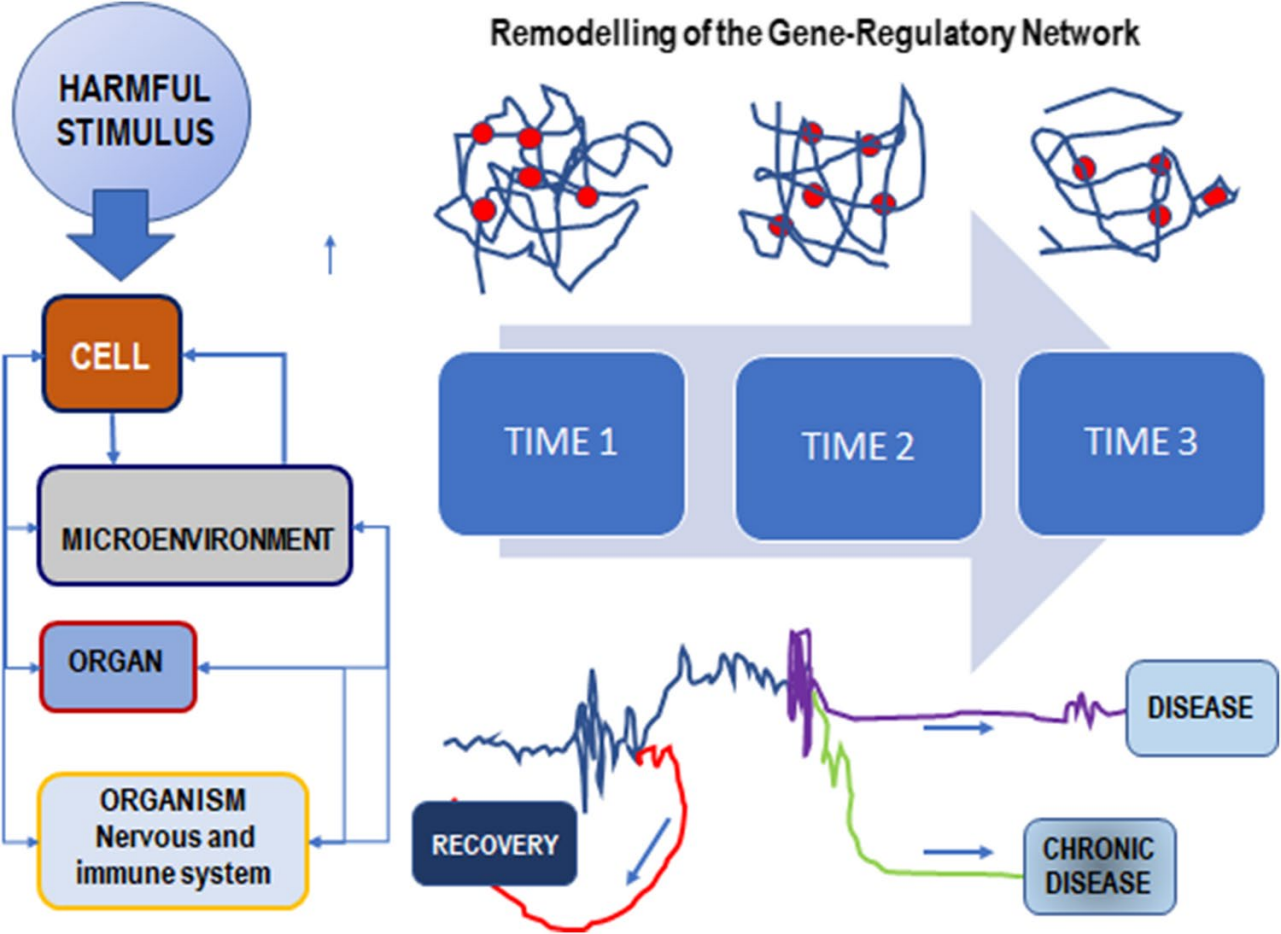
Disease as a historical, dynamical process. Most of harmful stimuli (exogenous toxicants, microbes, metabolic factors, radiation, etc.) hit different kind of cells and tissues, as only very few pathogenic cues interact with a single cell type. The response can be appreciated at both local and organismal level, involving the participation of many different tissues and structures. Overall, this entrenched cooperativity contributes to reshaping the Gene Regulatory Network as well as several biochemical pathways. The entire process proceeds across different bifurcation points (A, B) displacing itself through different attractors (i.e., phenotypic states), before reaching a stable “disease-state”

This premise carries additional consequences. Diseases — all together with their “causative” targets — are usually recognized by late-appearing manifestations. However, disease development entails several time-distributed steps, and specific treatments should be put in place at each distinct phase in which a specific rewiring of the Gene Regulatory Network is likely to occur (Fig. 1). Moreover, diagnostic parameters and putative causative factors are frequently associated with the steady state of the disease. This approach involves the obvious risk to consider a late-emerging symptom/target as the driver-causative element of the pathogenic process, while discarding early, critical signs.

Definitely, current PM-based treatments are unable to cope with such an overwhelming complexity, and their acknowledged failure in curing cancer cannot be viewed as an unannounced surprise [61] [63]. Consequently, “we overdiagnose, overtreat, and overpromise, with high costs and without clear benefits” [62].

## Reconsidering the concept of human disease according to a systems biology approach

The reductionist model of disease overlooks the relevance of multifactorial etiology of the disease and underestimates the robustness and resilience of the (pathologic) phenotype, especially when pharmacologically perturbed. For instance, single-gene knockout or complete silencing has shown little contradictory or even null effect on the pathophenotype [99]. Inhibiting a selected pathway can be insufficient in controlling the “corresponding” biological function, as the network can switch toward alternative in response to changing requirements of the context in which the system belongs [100, 101]. The switch occurs at specific “tipping points” where the system can enter into previously “unknown” attractors, thus acquiring “unexpected” features, including the resilience to a wide range of perturbing factors [102]. It should be stressed that at the bifurcation point, we usually observe an increase in the fluctuation of several parameters. Fluctuations are critical for enzymes to work, for a receptor to switch between states, and for the chromatin to express the right protein at the right time [103]. Overlooking these fluctuations will likely affect the identification of those states in which the system affords the choice in between different cell fate commitment [103]. These considerations may have huge consequences, given that the identification of such parameters would allow in recognizing those targets that are instrumental in driving transitions in the pre-disease state, thus performing true “preventive diagnostic.” Anticipating the transition from the pre-disease to the stable disease state represents a testable “personalization” of medical treatment. In other words, target recognition in the preventive perspective should shift from the disease to the highly dynamic and complex pre-disease state.

**Fig. 2:**
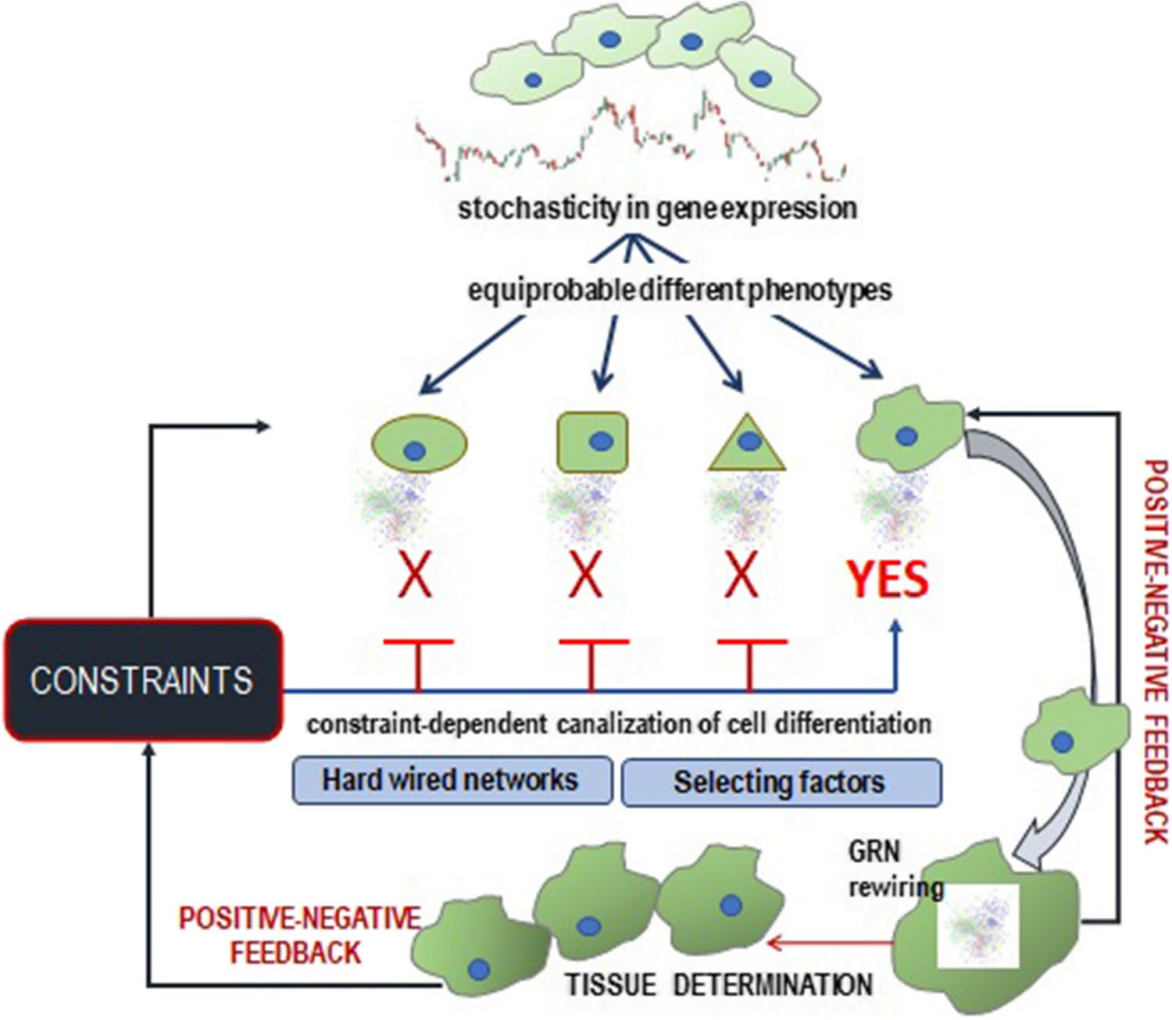
Constraints shape the gene expression pattern. Intrinsic stochasticity in gene expression pattern is constrained by internal/external factors that canalize the overall activity toward distinct phenotype configurations, by which cells and tissues differentiate. Stochasticity on gene expression can provide different phenotypes, all of which are compatible with the same genotype. However, subtle changes in physical and biochemical constraints — mostly provided by the microenvironment or coming from higher levels of organization (tissues, organ, etc.) — can “select” and “shape” only a specific phenotypic architecture. Once that phenotypic fingerprint has been chosen, then the overall system will set the genomic activity into a featured, stable configuration

The contribution of internal or environmental constraints in “driving” such transitions is mandatory, as they represent additional “causative factors” [104]. The emergence of a specific network associated with the time-dependent state of the disease process can be properly ascertained only if the specific microenvironmental field is *concurrently* contemplated. Genetic regulatory networks and the context-dependent constraints are tightly intertwined and therefore a successful therapeutic strategy should embrace all of them if the aim is properly to cure the patients, and not only “to fix” a “singled out” pathway [105]. Indeed, constraints exert a mandatory function in driving the systems toward distinct attractors, i.e., specific phenotypes recognizable by a different architecture of its Gene Regulatory Network (Fig. 2).

A comprehensive approach to this problem would enable in providing a complex network structure, constituted by modular sub-systems, whose (non-linear) interaction will drive the organism response toward emergent properties, i.e., disease or health. Therefore, human disease needs to be conceptualized as an “emergent property” of the human body [106]). Overall, these considerations ask for revisiting the concept of illness on which any personalized medicine should deal with. Systems biology may help in establishing a new model, able to integrate different “causative” factors, distributed across different scales (from molecules to organs) and times (from the early life to the present). Within this framework, response parameters should be provided by the overall system estimate, rather than on singled-out molecular target [107]. In other words, disease should be conceptualized as a non-linear dynamic process displaying classical features of complex systems, including resilience, sensitivity to initial conditions, and multi-attractor accessibility. Transition from different states — healthy, high-risk conditions, pre-disease, and disease states — has been documented and modeled in some instances [108, 109]. Namely, the existence of “critical period” during the lifespan (especially during early life) has been highlighted by studies that have identified a strong link between those periods and the appearance of different diseases in the adult period [110, 111]. During the development, organs and systems of the body go through “critical” periods, in which their sensitivity to internal and environmental perturbations is dramatically amplified [112]. While it is intuitive that such a plasticity — i.e., the capacity to dynamically respond to surrounding stresses by shaping morphology and functions — is an advantage from the evolutionary perspective, nonetheless such a capability exposes the organism to unwarranted risks. Indeed, when the system experiences important perturbations, even pathological states can arise and eventually be maintained if they become reliable, adaptive issues.

Briefly, the physiological system can travel across that metaphorical landscape (remnant of the Waddington’s approach) [113], accessing different attractors, depicted as different physiological/pathological states — a normal state, a pre-disease state, a disease state — in which the disease can alternatively move toward progression or healing. Identification of these bifurcation points could help in understanding the meaning of the overall process and in managing it toward beneficial outcomes [114]. Finally, identification of biomarkers (“early-warning signals”) indicating an imminent bifurcation or sudden deterioration before the critical transition occurs can help in planning an appropriate management of the disease or the pre-disease state, thus providing the “preventive” strategy with an entirely new meaning [115].

## Conclusions and expert recommendations

The practice of medicine is currently undergoing a paradigm shift, aimed at treating a disease by identifying the specific fingerprint displayed by the illness in an individual; insofar each patient can be featured through a personalized data-driven approach, as that allowed by the convergence of big data and multi-omics approaches.

Such a task entails several ethical, social, and regulatory issues [116, 117]. As a consequence, several theoretical and methodological premises on which PPPM relies require to be reconsidered given that the complexity of a bewildering number of extragenomic factors undermines the predictability of PM models based prevalently on genomic data [118, 119].

For most common diseases, hundreds of genetic risk variants with small effects have been identified, and it is hard to establish an unambiguous picture of who is really at “risk” and for “what.” Definitely, no convincing evidence allows in recognizing a one-to-one correspondence between genomic alterations and widespread, complex diseases for which “traditional” markers are much better predictors than extensive DNA data sets [118]. Notice that inappropriate and exaggerate expectations in PPPM promises will easily turn out into false hope. People believing not to be at risk after being reassured having performed a “genome-based test” will probably dismiss appropriate behaviors and health care precautions; on the contrary, people “informed” to be at high risk shall probably endorse a resigned attitude, therefore discarding medical controls and treatments [120].

Despite PPPM missed its promises, we yet continue to overinvest our hope (and money) in genomic-based approaches. Yet genetics cannot deliver unaffordable expectations — as recognized even by PM advocates during the recent Covid-19 pandemics [121] — given that genes do not constitute that “privileged” level of causation the reductionist medicine was searching for establishing a deterministic biological model [122]. In simple words, we actually lack the minimal facts needed to support the hope that genetics can be a key to realizing that vision. This statement implies we should orchestrate a stratified and multilevel diagnostic and treatment approach [123].

A more prudent reappraisal of PPPM has evidenced the paradox behind this approach. In principle, PM-based treatments are themselves more precise than standard chemotherapeutic protocols although the clinical evidence supporting the benefits of these therapies is often considerably less precise and still awaits sound confirmation [124]. Paradoxically, clinical trials designed to assess PPPM efficiency demonstrated a lack of precision in respect to conventional trials [125]. Namely, these studies comprise several treatment arms in which far fewer patients than in conventional phase III investigations are generally enrolled. It is quite disturbing that these patients are usually not randomized owing to the difficulties in determining a sufficient number of eligible patients or in planning a proper control treatment. Furthermore, results are biased by the limited choice of end-points (usually confined to the overall response rate), thus undermining the trials’ validity.

Secondly, to “extract” useful knowledge from a complex system, one must focus on the right level of description [126]. No doubt that the disease is an emergent phenomenon involving the organism and not limited to molecules or cells. Thereby, any attempt to establish a precision-based treatment should look at the “system,” instead of focusing on single molecules or pathways. To sum up, despite undeniable performances recently performed in the context of predictive approaches, targeted prevention, and personalization of medical care (PPPM/3PM), several aspects are waiting for innovative solutions [127].

In detail, treatments should be tailored according to the “timing-frame” of each condition. This approach can help in detecting those critical transitions through which the system can access different attractors leading ultimately to diverse outcomes — from a pre-disease state to an overt illness or, alternatively, to recovery. Identification of such tipping points can substantiate the predictive and the preventive ambition of the PPPM/3PM.

Namely, analyses performed on liquid biopsies by means of mass spectrometry‐based technologies [128] have allowed the identification of a wide array of biomarkers and of their specific pattern of expression that both can provide valuable diagnostic information for the identification of overt disease, its preliminary stages, and ultimately the very initial (reversible) conditions. Thorough investigation carried out on liquid biopsy specimens can be instrumental in drawing precise phenotyping, i.e., a multi-omics fingerprinting — that, in turn, would allow the creation of tailored treatment algorithms, thus providing the most optimal clinical approach for personalized, predictive, and preventive medical service [129].

This perspective implies that we should shift from targets to processes and identify a multi-target array — entailing different mechanisms of action — as suggested by the network polypharmacology approach. Identification of such tipping points can substantiate the predictive and the preventive ambition of the PPPM/3PM.

Namely, analyses performed on liquid biopsies by means of mass spectrometry‐based technologies [128] have allowed the identification of a wide array of biomarkers and of their specific pattern of expression that both can provide valuable diagnostic information for the identification of overt disease, its preliminary stages, and ultimately the very initial (reversible) conditions. Thorough investigation carried out on liquid biopsy specimens can be instrumental in drawing precise phenotyping, i.e., a multi-omics fingerprinting — that, in turn, would allow the creation of tailored treatment algorithms, thus providing the most optimal clinical approach for personalized, predictive, and preventive medical service [129].

This perspective implies that we should shift from targets to processes and identify a multi-target array — entailing different mechanisms of action — as suggested by the network polypharmacology approach [130]. Modulation of processes implies we should be able to “redraw” the disease-related landscape, favoring the system displacement from pre-clinical state or true disease-states toward healing pathways. Disease could eventually be “reverted,” involving also a “reprogramming” of the gene-regulatory network, as proposed in some carcinogenic models [131–133]. Indeed, as cancer can be successfully “reverted” through the modification of the dynamical cross talk with its microenvironment, interactive cell-stroma networks must be recognized as a target for pharmacological intervention [60, 134, 135]

How should we investigate the evolution of such a complex system over time? We know that distributed non-linear network systems are hardly mathematically tractable when matched with simple feedback systems, usually described by means of control theory. Disease response is habitually simplified by describing changes in a single (or a few) parameter. Instead, we have to move from target-related parameters to system parameters, which could capture those modifications that could likely impact on the whole systems dynamics [136]

At the core of this new strategy, a relevant role is sustained by multi-omics data, which can likely help in moving from a genome-centered to a phenome-centered research practice. However, an unusual effort is required to conjugate different high-throughput analytical methods, data collection, and network analysis reconstruction (eventually involving innovative Artificial Intelligent tools) to recognize critical phases and relevant targets [137]. Noteworthy, this approach could help in allowing a patient stratification according to a very different perspective [138, 139]

A relevant case in point is constituted by metabolomics studies that — in principle — can satisfy those needs. Metabolites fluctuations usually amplify subtle modulation of the genome/proteome networks over time and metabolomics can describe changes in complex systems dynamics as well as its “adaptive” phenotype in presence of a wide range of perturbations [140–142], thus providing a new basis for establishing a proper PPPM/3PM framework. Noticeably, a striking correlation can be established between metabolomics and proteomic changes, and both can play a driving role during cell/tissues phenotypic transitions. Moreover, proteomic changes can be viewed as the ultimate output of several processes — transcriptomic variations, splicing, post-translational modifications, translocation/re-distribution, spatial conformation, and pathway-network systems — that collectively might contribute to clarify the mechanisms of a disease and to recognize therapeutic targets [143].

Future interventions should aim to find the way to “modulate” the metabolomic fingerprint of a disease in a “precise” and efficient fashion, instead of searching for hypothetical “causative target” and “magic bullets.” Some pioneering approaches demonstrated that such an approach could successfully recognize subsets of patients within the same disease, characterized by different pathophenotypes and distinct activated pathways that can be successfully targeted with very different treatments [144]. This selection is performed through a systems biology approach and does not rely on single targets as it focuses on the overall behavior of the system under scrutiny.

## Data Availability

All quoted articles are available at request.

## Abbreviations

PPPM/ePM: Predictive, Preventive, and Personalized Medicine
PM: Personalized medicine
SMT: Somatic Mutation Theory
TOFT: Tissue organization field theory
NCI-MATCH: National Cancer Institute–Molecular Analysis
